# A Case of Primary Central Nervous System Lymphoma Mimic Neuromyelitis Optica

**DOI:** 10.1515/tnsci-2020-0005

**Published:** 2020-02-18

**Authors:** Xixi Sheng, Mingwei Xu, Xia Li

**Affiliations:** 1 Department of Neurology, The First Affiliated Hospital, College of Medicine, Zhejiang University, Address: No. 79 Qing-Chun Road, Hangzhou, Zhejiang 310003, PR China

**Keywords:** Lymphoma, Neuromyelitis optica, primary central nervous system, case report, PCNSL

## Abstract

Primary central nervous system lymphoma (PCNSL) is rare. And the symptoms of PCNSL are atypical, it is extremely easy to be misdiagnosed as other diseases. However, early treatment is crucial which is requesting early diagnosis. We report a case of a 47-year-old man who was initially diagnosed as neuromyelitis optica (NMO) on the basis of clinical findings, slightly high Aquaporin4 (AQP4) (1:10) and high signals of magnetic resonance imaging. Though his symptoms progressively improved after steroid pulse treatment, but worse when steroid was decreased to 40 mg per day. We considered the patient should be diagnosed as PCNSL. After the examination of magnetic resonance spectroscopy (MRS) and positron emission tomography (PET), the results indicated PCNSL was most possible. Therefore we gave him stereotactic biopsy of deep of supratentorial, which showed non-Hodgkin malignant B-cell lymphoma.

## 1 Introduction

Primary central nervous system lymphoma (PCNSL) is rare [1]. The symptoms of PCNSL are atypical as was easy to be misdiagnosed as other diseases [2]. There are some cases of PCNSL that are misdiagnosed as other diseases, such as Parkinson’s disease, myasthenia and multiple sclerosis [3]. There are some reasons. First, PCNSL represents less than 2% of all brain neoplasms and 1%–2% of malignant lymphomas. Most are B cell non-Hodgkin lymphoma (NHL), which may present as single or multiple lesions involving the eye, leptomeninges, or brain parenchyma. Localization primarily in the brain stem occurs in 3% of PCNSL, and most are T cell in origin [4]. In the present work, we present a rare case of primary central nervous system lymphoma which mimic neuromyelitis optica.

## 2 Case Report

A 47-year-old man presented with a 1-week history of neck pain and 2-day history of shoulders pain. He showed upper limb weakness and under the right chest numbness. However, He had no history of smoking and drinking, as well as persistent weight loss. He also denied the history of hypertension and diabetes.

On neurological examination, the patient was found to have a shallow of the left frontal line and nasolabial groove and hypaesthesia under T4 the right chest. Muscular tension was normal, but upper limbs muscle strength was 4-/5 and lower limbs muscle strength was 5-/5. Tendon reflexes were normal and both Kernig’s and Brudzinski’s signs were negative, but bilateral Babinski signs and left Hoffmann sign were positive. Analyses of viral antibodies, syphilis serology, and human immunodeficiency virus antibodies were negative. Lumbar puncture showed normal intracranial pressure. In the CSF findings (Table 1), tumor cells were not observed. Red cells were 10/ul and nuclear cells were 220/ul, however, the classification flow cytometry of cerebrospinal fluid leukemia had not found abnormal differentiation of cells. The protein level was slightly elevated (91 mg/ dL), however, chloride (127 mmol/L) and glucose (3.32 mmol/L) were normal. Oligo-clonal bands were not present. Other markers of autoimmune diseases, such as antinuclear antibodies, extractable nuclear antigen, and antineutrophil cytoplasmic antibodies, were all negative. However, some cytokines were slightly high (IL-2(0.1pg/ ml), IL-6 (67.75 pg/ml), TNF-α (50.66pg/ml), others (IL-2, IL-10, IL-17 and IFN-γ) were normal. Serum anti-AQP4 antibodies were slightly positive (1:10) on April 6^th^ 2016,

**Table 1:** The results of the CSF findings

Examination	CSF/serum	Results
Tumor cells	CSF	Negative
Red cells	CSF	10/ul
Nuclear cells	CSF	220/ul
Protein	CSF	91mg/dL
Chloride	CSF	127mmol/L
Glucose	CSF	3.32mmol/L
Oligo-clonal bands	CSF	Negative
Antinuclear antibodies	serum	Negative
Extractable nuclear antigen	serum	Negative
Antineutrophil cytoplasmic antibodies	serum	Negative
Il-2	serum	0.1pg/ml
Il-6	serum	67.75pg/ml
TNF-α	serum	50.66pg/ml
Anti-aqp4 antibodies	serum	Positive

**Figure 1:**
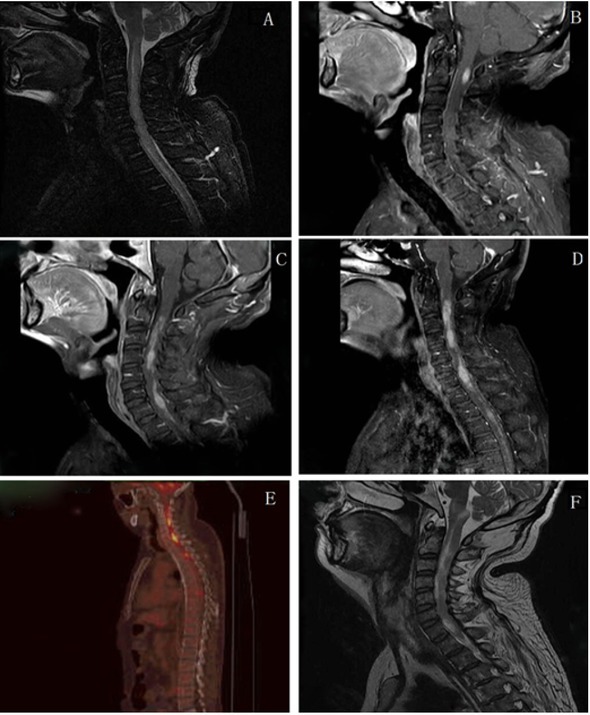
MRI of cervical spinal cord demonstrated multiple lesions with occupied in C2-T1 spinal and brain stem. (March 26^th^) A: Sagittal T2 weighed showed enlargement of the cervical spinal. (April 1^st^) B and C: Sagittal T1 enhanced imaging showed the enhanced lesion in C2-T1 level and brain stem and slightly enlargement of the cervical spinal. (April 29^th^) D: Sagittal T1 enhanced imaging showed multiple abnormal enhanced lesion in C2 below and in the intrathoracic spinal and brain stem, compared with 2016-04-01 images, lesions increased. (May 19^th^) E: Positron emission tomography (PET) showed obvious increased 18-Fludeoxyglucose (18F-FDG) uptake in the C1-C7, T2-T3, T7 spinal cord. (July 20^th^) F: Sagittal T2 showed multiple abnormal enhanced lesion in C2 below and in the intrathoracic spinal and brain stem, compared with 2016-04-29 images, lesions decreased.

**Figure 2:**
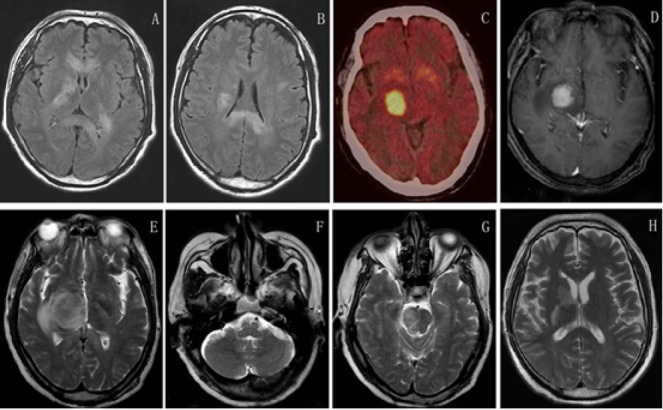
Magnetic resonance imaging revealed that right basal ganglia, pons, cerebellar peduncles with abnormal signals.（April 5^th^） A and B: Magnetic resonance scans showed bilateral thalamus, periventricular, basal ganglia, pedunculus cerebri with hypersignals using fluid attenuated inversion recovery.（May 19^th^）C: Positron emission tomography (PET) showed a slightly high enhancement in the right basal ganglia and right pons, about the size of which is 2.1*2.0*2cm and the maximum SUV value is 23.6. (May 23^th^) D, E, F and G: A month and a half later, axial T1 and T2 magnetic resonance imaging revealed that right basal ganglia, pons, cerebellar peduncles with hypersignals, and lesions were larger than the former, even involved in bilateral thalamus. This enlargement would be suggestive of a neoplastic process. (July 20^th^) H: Axial T2 magnetic resonance imaging revealed that right basal ganglia, pons, cerebellar peduncles with abnormal signal, compared with 2016-05-23, lesions decreased.

CSF: cerebrospinal fluid

which was consistent with the diagnosis of NMO. Magnetic resonance imaging of the cervical spinal cord revealed multiple lesions occupied in C2-T1 spinal and brain stem, high in T2 weighed, enlargement of the cervical spinal and evident enhancement in the enhanced imaging **(Figure1)**. Magnetic resonance scans showed bilateral thalamus, periventricular, basal ganglia, pedunculus cerebri with hypersignals using fluid-attenuated inversion recovery (Figure 2 A and B). Combination of the clinical symptoms, neurological examination, laboratory examination and magnetic resonance imaging, the initial diagnosis was considered as neuromyelitis optica with steroid pulse treatment (methylprednisolone 1g intravenous injection per day) and immunoglobulin. After steroid treatment, his symptoms progressively improved and he returned home.

**Figure 3:**
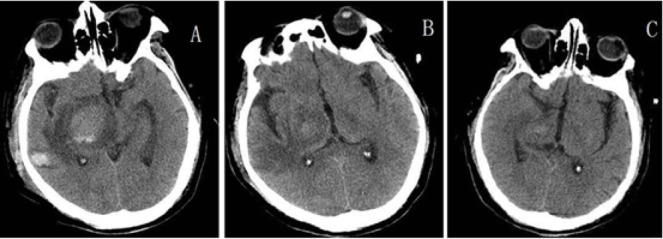
CT scan showed the right basal ganglia and right temporal lobe hemorrhage with edema (May 29^th^) A：Brain CT scan showed the right basal ganglia flake slightly higher density, CT value of about 32U, surrounding low-density, right temporal lobe low-density, the intracranial brain parenchyma density was not significantly abnormal changes. Midline slightly shifted to the left. (June 14^th^) B and (June 27^th^) C: Brain CT scan image is about similar to the image in 5.29, bleeding fade, edema reduced.

**Figure 4:**
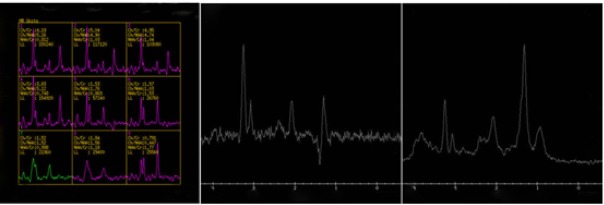
The MRS showed a high resonance of free lipids and Cho, a low resonance of NAA, and a high Cho/NAA ratio (4.8-5.1) .

**Figure 5:**
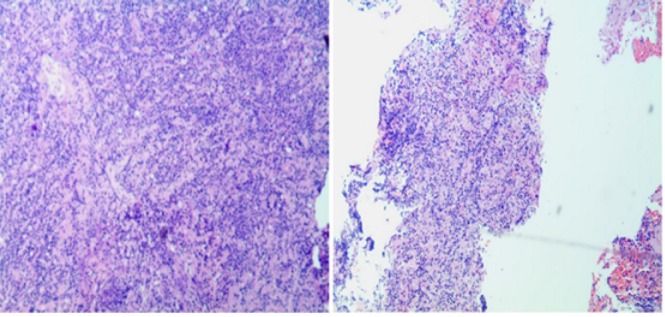
Pathology of the brain showing:(Right basal brain tissue): Non-Hodgkin's lymphoma, Diffuse large B cell type. CD3 (-), CD20 (+), 6-1 CD79a(+), CD30(Ki-1)( -), CD5(-), CD10(-), BcL-2 (80%+), Bcl-6 (-), ALK(-), MUM1 (+), Ki-67 (90%+++), PAX-5 (+), Myc (40%+), CyclinD1 (-).

However, when steroid was decreased to 40 mg per day, his symptoms worsened and he was admitted to our hospital again. He presented with limbs weakness, spirit weakness and vision loss in the left eye in 2 months. His limb muscle strength gradually declined to grade 0 2 months later. Review serum anti-AQP4 antibodies were negative on May 20th 2016. Magnetic resonance imaging revealed that right basal ganglia, pons, cerebellar peduncles with hypersignals, and lesions were larger than 2 months ago (Figure 2 C, D, E and F). The MRS showed a high resonance of free lipids and Cho/NAA ratio (4.8-5.1), lactates were also visible, which was in favor of a tumoral process (Fig4). Positron emission tomography (PET) showed a slightly increased 18-Fludeoxyglucose (18F-FDG) uptake in the right basal ganglia and right pons and obviously increased uptake of 18F-FDG in the affected brain, the maximum SUV (Standardized Uptake Value) value is 23.6, indicating PCNSL possible (Figure1E and Figure 2C). (May 27th) Stereotactic biopsy of deep of supratentorial was performed and immunohistochemical analysis showed that the brain tissue was infiltrated with multiple foci of heterotypic cells with CD20 (+), 6-1 CD79a (+), Ki-67 (90%+++), BcL-2 (80%+) (Figure 5), suggestive of non-Hodgkin malignant B-cell lymphoma. (May 29th) After a stereotactic biopsy, the patient appeared unconscious. Arterial blood gas showed partial pressure of carbin dioxide (pCO2) elevated. After endotracheal intubation and mechanical ventilation, the patient’s pCO2 gradually decreased and the patient turned to awake. Neurosurgical consultation considered: combination of symptoms and CT scan which showed the right basal ganglia and right temporal lobe hemorrhage with edema to consider (Figure 3A), first consider as piercing bleeding. According to these results, the patient was given chemotherapy. The patient was not given radiotherapy, because he had a severe lung infection. Methotrexate was used at a high dose (5 g/m2) and the hypaesthesia plane was down to T2 the right chest. Then he has given rituximab 600 mg and the second course of Methotrexate at a high dose (5 g/m2). After the third course of methotrexate cycle, his symptoms were not improved further. He was given intrathecal injection of MTX 15mg + Ara-e 50mg + DXM10 mg. Later, CT scan image, compared to 2016-05-29 CT image, bleeding fade, edema reduced, which also supported the previous diagnosis of piercing bleeding (Figure 3B and C). However, Magnetic resonance imaging of the brain and spinal cord showed that the lesions of the right thalamus and midbrain were decreased (Figure1F and Figure2H) and oblongata and the cervical cord had no change, his symptoms still were not improved. Unfortunately, 9 months after the first admission, he died of the disease.

**Ethical approval**: The research related to human use has been complied with all the relevant national regulations, institutional policies and in accordance the tenets of the Helsinki Declaration, and has been approved by the authors’ institutional review board or equivalent committee.

**Informed consent**: Informed consent has been obtained from the family members of the patient included in this study

## 3 Discussion

Primary central nervous system lymphoma (PCNSL) is rare clinically without the exact incidence rate [1]. PCNSL is not firstly considered in most of the clinical cases because we often diagnose diseases as single and common diseases. In the usual case of neurologists, PCNSL are usually the last to consider. Second, PCNSL may present with all symptoms, such as limb weakness, hemianopsia, hypersomnia, and binocular vertical diplopia. Though typical optic neuromyelitis showing acute severe long myelitis and bilateral or simultaneous symptoms, some patient’s symptoms may slowly progress from one side to the other. Symptoms can prompt us for some disease, but not the only criteria for diagnosing the disease. So we diagnose diseases with a combination of symptoms, laboratory tests and radiographic. Third, some laboratory tests may be also prone to other diseases. Although the NMO is also not frequently diagnosed clinically with the incidence of 2.56 out of 100,000 persons [5]. NMO-IgG has been reported to be a novel serological marker for NMO [6]. But there have been several case reports of NMO-IgG coincident with cancer, suggesting NMO-IgG possibly being a paraneoplastic marker [7, 8, 9]. Neoplastic cells may provide the antigen initiating an aquaporin-4 immune response. Tumor cells express, as onconeural antigens, proteins that are normally expressed by mature neurons, glia, or muscle. Cancer-directed immune responses initiated by those antigens have the potential to target autoantigens in the nervous system [10]. In this case, AQP4 serum-positive(1:10) may be caused by PCNSL rather than NMO. But it is not denied that AQP4 serum-positive can help diagnose NMO. Finally, the radiographic examination maybe not typical in some cases. The radiographic of the patient was over three vertebral segments, slightly high in T2 weighted and enlargement of the cervical spinal, which is not typical. The radiological spectrum of the CNS lesion in NMO is very wide. Longitudinally extensive transverse myelitis, over 3 vertebral segments, and optic neuritis, extensive and involving posterior segment, are essential findings for the diagnosis [11]. So the current case is an unusual presentation of primary brain stem B cell non-Hodgkin’s lymphoma, which was initially misdiagnosed as NMO on the basis of clinical findings, slightly high AQP4 and magnetic resonance imaging that accorded with the characteristic imaging of NMO. This case needs to attach clinical neurologists great attention to some points: when we diagnose a patient as NMO, we also should differentiate from PCNSL. We need other more effective methods to help differentiate NMO and PCNSL. By assessing some metabolic changes for many pathological processes, localized Proton Magnetic Spectroscopy (MRS) has been successfully used as a more sensitive and non-invasive tool for differential diagnosis [12]. In some cases, after the steroid pulse treatment, the symptoms are still progressive, we could suggest patient to have a test of MRS. If the MRS of the patient shows a high resonance of free lipids and visible lactate, especially a high Cho/NAA ratio, PCNSL should be considered [12]. Except for MRS, it showed that the CSF sIL-2R level is a biomarker that can differentiate CNS IDDs (inflammatory demyelinating diseases) from CNS lymphoma. CSF sIL-2R is founded higher than in the patient of PCNSL than of CNS IDDs [13]. NMO is one of CNS IDDs, so CSF sIL-2R maybe also can help differentiate PCNSL from NMO. However, the final diagnose is needed a stereotactic biopsy. Immunohistochemical analysis not only can help diagnose the patient as PCNSL, but also can certain the pathological type. All these tests can help differentiate CNS lymphoma from NMO, but most important thing is that we have a concept that we should differentiate a common diagnose from PCNSL and we know the best time when we should give the patient the examination of MRS, CSF sIL-2R and stereotactic biopsy.

There are several possible causes of this patient’s intracranial hemorrhage. First, the most likely cause of intracranial hemorrhage in this patient is puncture bleeding according to the history of stereotactic biopsy and the CT image after two weeks. And it is also based on the site of intracranial hemorrhage in the puncture site. Second, the patient’s intracranial hemorrhage cannot rule out the possibility of tumor necrosis bleeding because of the PCNSL invasion of the basal ganglion. Intratumoral hemorrhage may be seen, which is rare in the immunocompetent patient [14]. This patient’s analyses of viral antibodies, syphilis serology, and human immunodeficiency virus antibodies were negative. The possibility of intratumoral hemorrhage is small. Third, it may also be due to thrombocytopenia caused by chemotherapy drugs, such as Methotrexate, which induce intracranial hemorrhage. However, this patient tested blood routinely, so this possibility is also small.
